# Orbital Compartment Syndrome: How a Young Man’s Vision was Saved by the Timely Actions of an Emergency Medicine Physician

**DOI:** 10.7759/cureus.5057

**Published:** 2019-07-01

**Authors:** Jessica Houck, Rohan Mangal, Chrissy Vandillen, Latha Ganti, Bryan C Sleigh

**Affiliations:** 1 Emergency Medicine, University of Central Florida College of Medicine / Hospital Corporation of America Graduate Medical Education (HCA GME) Consortium, Kissimmee, USA; 2 Emergency Medicine, John Hopkins University, Baltimore, USA; 3 Emergency Medicine, St. Cloud Regional Medical Center, St. Cloud, USA; 4 Emergency Medicine, Envision Physician Services, Orlando, USA; 5 Emergency Medicine, Mercer University School of Medicine, Macon, USA

**Keywords:** orbital compartment syndrome, lateral canthotomy

## Abstract

In the following case presentation, a young man who incurred orbital compartment syndrome (OCS) from physical trauma significantly improved from timely lateral canthotomy. Lateral canthotomy is recommended to be performed as soon as possible to avoid permanent vision loss, which is the most feared complication associated with orbital compartment syndrome. This procedure completely restored vision in the patient and permitted prompt discharge.

## Introduction

Orbital compartment syndrome (OCS) is an ophthalmic emergency that describes an increase in intraorbital volume and pressure, resulting in retinal ischemia [1,2]. Within two hours, patients may experience irreversible vision loss, unless the condition is quickly recognized and treated.

In situations of OCS, emergency physicians often do not have an ophthalmologist accessible to emergently treat patients [3]. To promptly treat this condition, lateral canthotomy is a relatively simple procedure that is recommended to prevent vision loss.

## Case presentation

**Figure 1:**
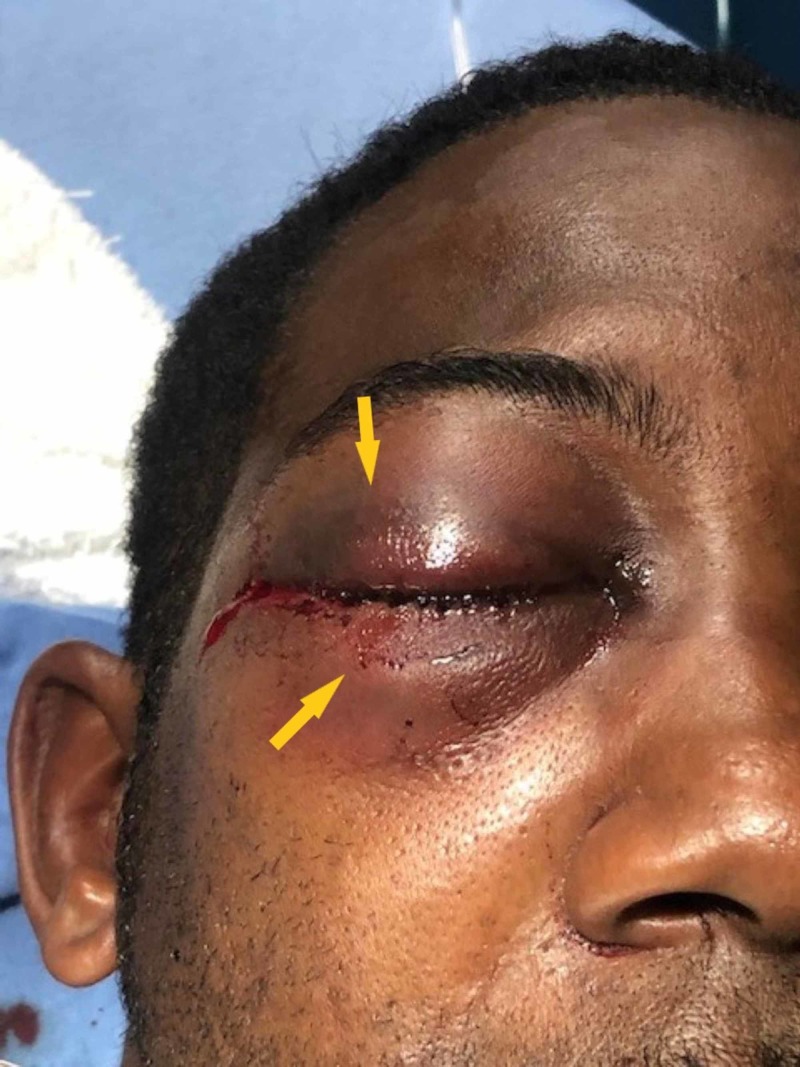
Patient with eyes closed, in position of comfort Arrows show periorbital swelling.

**Figure 2:**
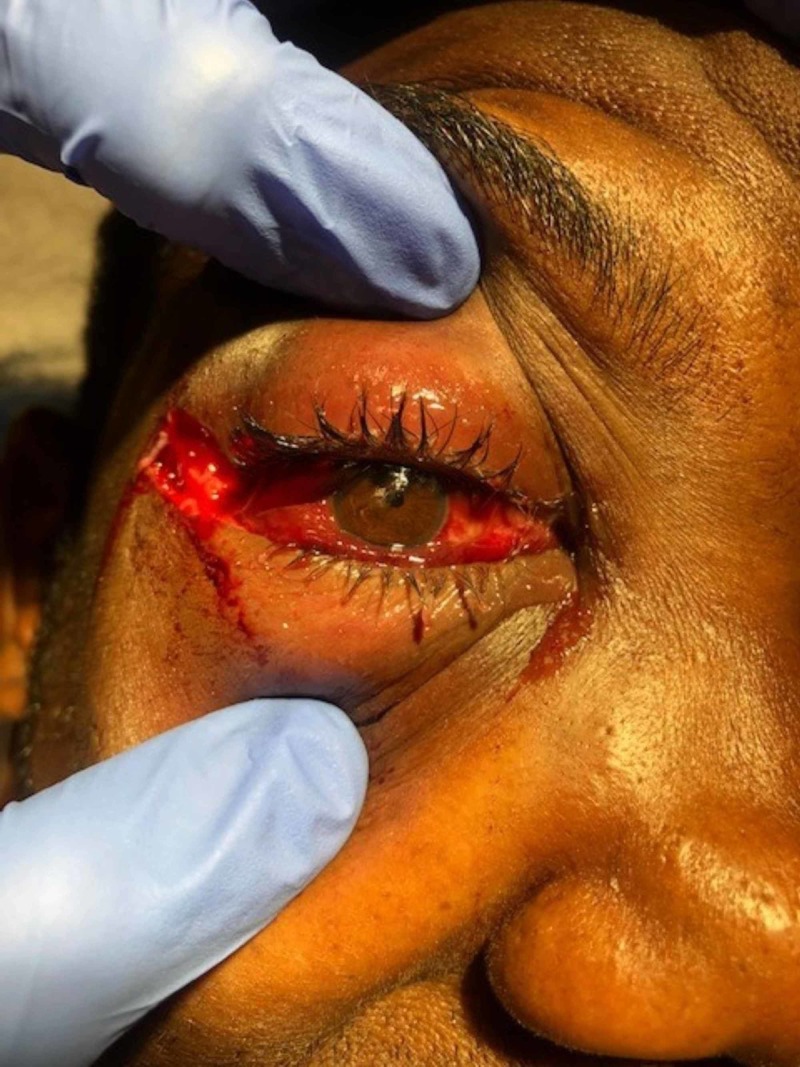
Patient with eye propped open by examiner

A 32-year-old male presented to an outside emergency department (ED) one hour after being physically assaulted, suffering from multiple blows to his face. On our exam, the patient had significant right periorbital swelling with proptosis (Figures 1-2).

**Figure 3:**
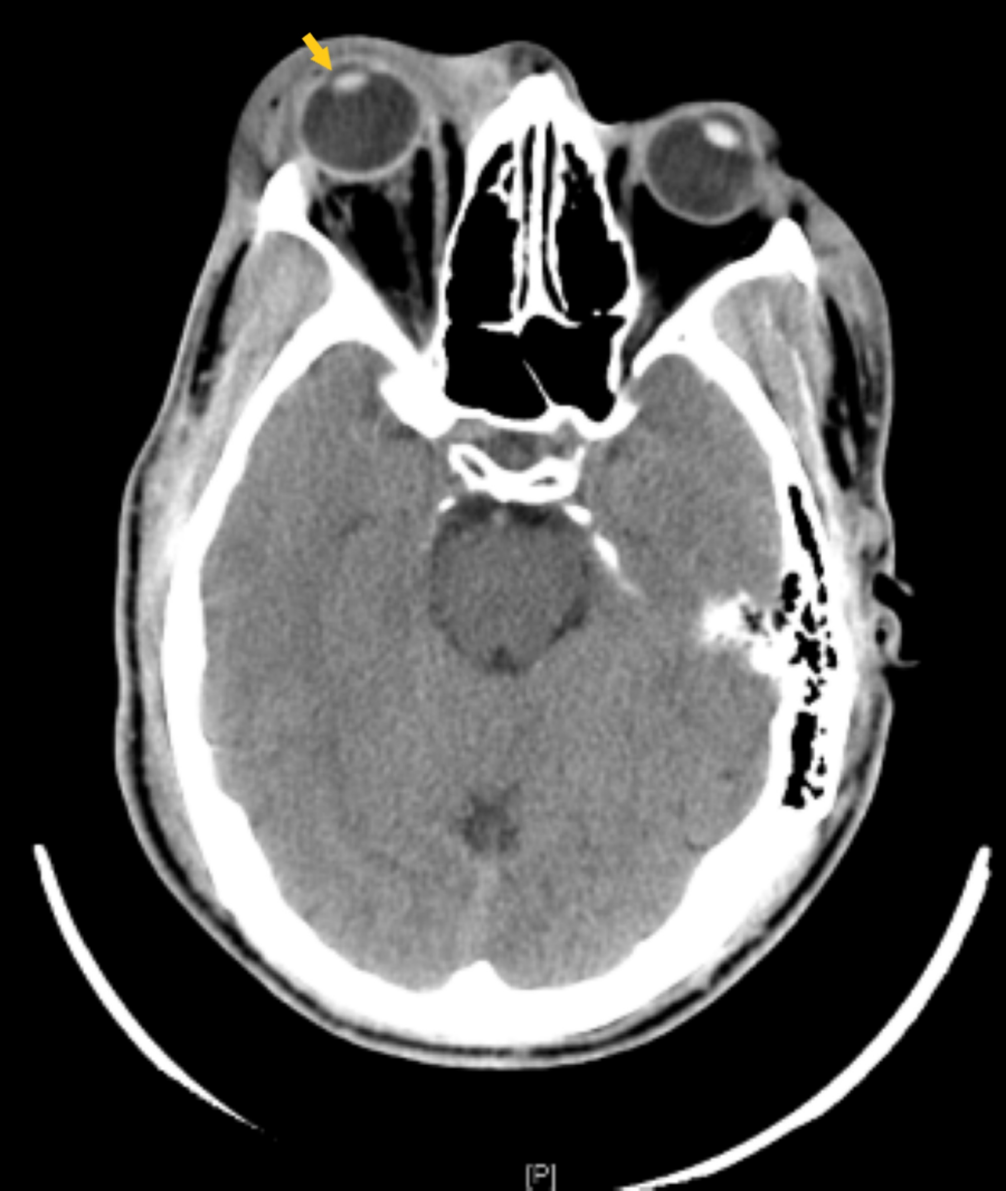
CT demonstrating right exophthalmos (arrow) with intraorbital fat stranding and abnormal caliber and density of the optic nerve sheath complex. Also seen is right preseptal subcutaneous soft tissue swelling and hematoma.

Computer Tomography (CT) scan of the head, orbits, and maxillofacial bones were obtained which revealed right exophthalmos with intraorbital fat stranding and abnormal caliber and density of the optic nerve sheath (Figure 3). The decision was made to transfer the patient to our facility for ophthalmology evaluation.

**Figure 4:**
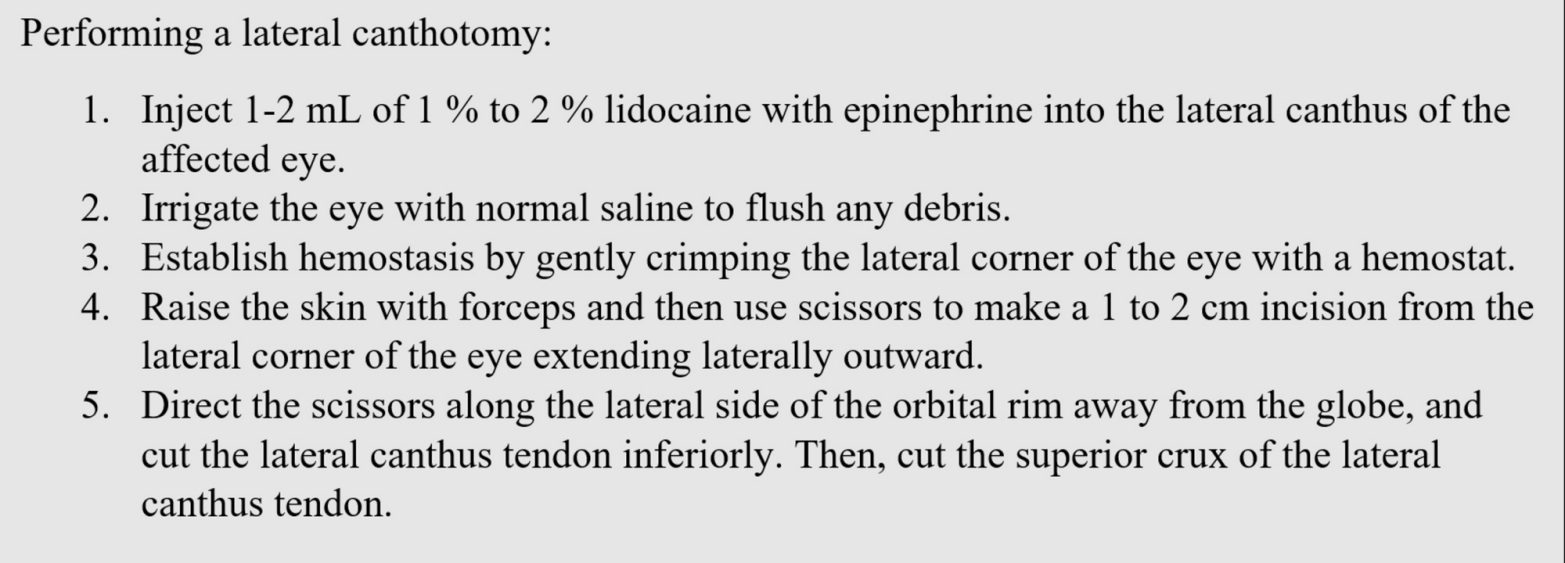
Technique for performing lateral canthotomy

Upon talking with the transferring physician, we learned that the patient had nearly complete loss of vision in the affected eye. Knowing that orbital compartment syndrome can cause permanent ischemic changes as quickly as within 60 minutes of onset, my attending physician requested that a lateral canthotomy procedure be performed prior to transfer. We were able to walk the transferring physician through the procedure (Figure 4).

**Figure 5:**
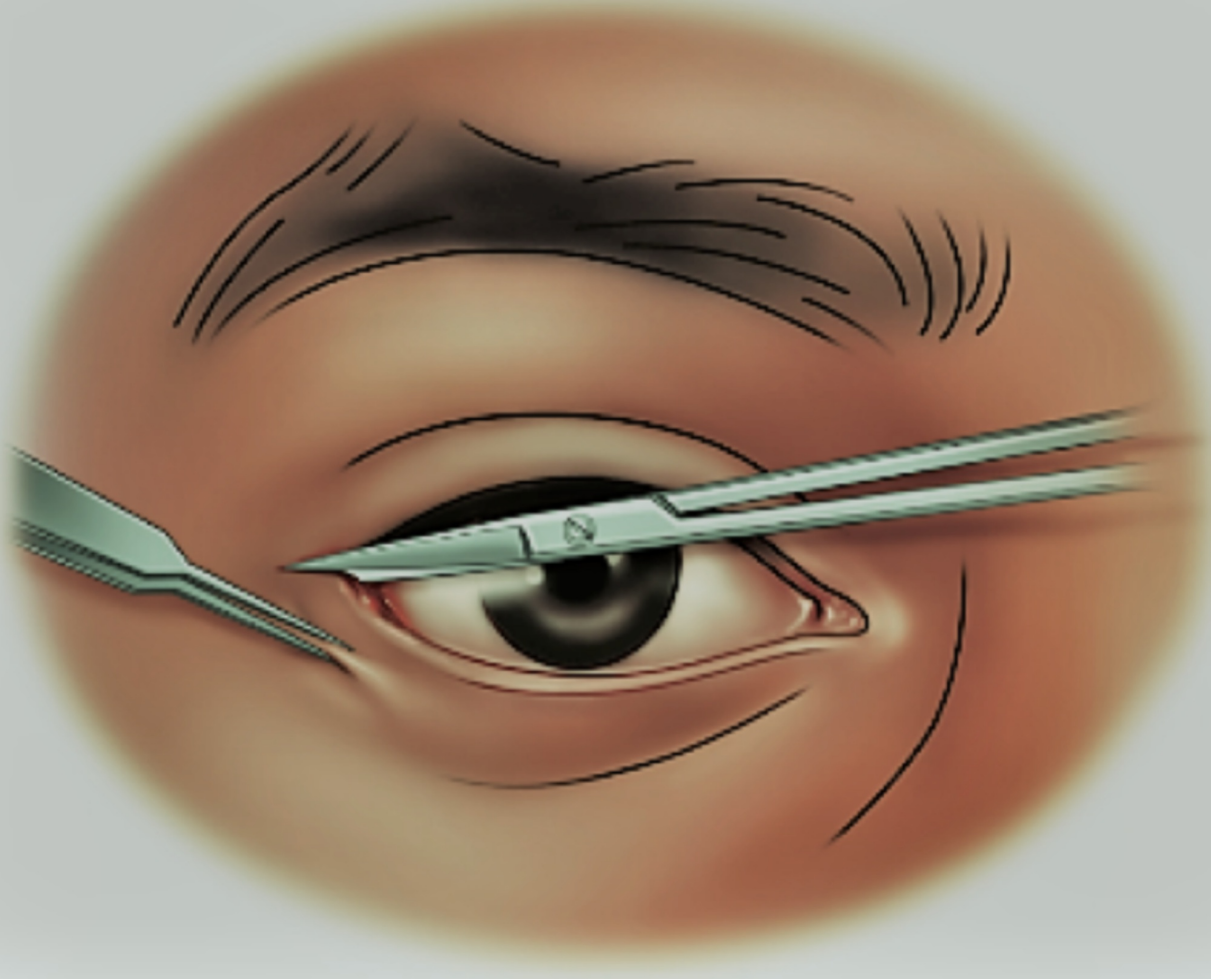
Cut for lateral canthotomy to reduce intraocular pressure [3]

A lateral canthal incision was made for lateral canthotomy (Figure 5). On the following day, the patient had complete restoration of his vision and was discharged home with ophthalmology follow up.

## Discussion

Patients with orbital compartment syndrome may experience symptoms such as a sudden onset decrease in visual acuity associated with pain, proptosis, ophthalmoparesis, and a relative afferent pupillary defect [4].

The indications for performing a lateral canthotomy include: intraocular pressure greater than 40 cm H_2_O, trauma or orbital cellulitis with vision loss, and exophthalmos. From observing improvement in this patient’s condition, it is emphasized that physicians should not wait for imaging to perform a lateral canthotomy, as permanent ischemic changes can occur within 60 minutes of onset.

The learning points are described as following: when performing a lateral canthotomy, start with the inferior canthal ligament; if this does not successfully reduce the proptosis then proceed with cutting the superior ligament as well. Repeat the intraocular pressure (IOP) measurement directly after the procedure.

In a sample of 190 emergency department physicians, 37.1% indicated they would comfortably perform a lateral canthotomy, while 95.7% recognized permanent vision loss as a potential consequence of orbital compartment syndrome [5]. This case aims to reaffirm the sight-saving impact and importance of a lateral canthotomy for OCS.

## Conclusions

Orbital compartment syndrome is an ophthalmologic emergency that requires immediate identification and treatment. Delaying treatment beyond one hour can put the patient at critical risk of permanent vision loss. A relatively simple procedure such as lateral canthotomy should be strongly considered as soon as possible to improve patients’ conditions.

## References

[1] Timlin HM, Bell SJ, Uddin JM, Osborne S (2019). Treatment outcomes of lateral canthotomy and cantholysis for orbital compartment syndrome. Br J Oral Maxillofac Surg.

[2] Rowh AD, Ufberg JW, Chan TC, Vilke GM, Harrigan RA (2015). Lateral canthotomy and cantholysis: emergency management of orbital compartment syndrome. J Emerg Med.

[3] Mahon BM, Desai BK (2016). Lateral canthotomy. Atlas of Emergency Medicine Procedures.

[4] Sun MT, Chan WO, Selva D (2014). Traumatic orbital compartment syndrome: importance of the lateral canthomy and cantholysis. Emerg Med Australas.

[5] Edmunds MR, Haridas AS, Morris DS, Jamalapuram K (2019). Management of acute retrobulbar haemorrhage: a survey of non-ophthalmic emergency department physicians. BMJ.

